# Non-Native *R1* Substitution in the S4 Domain Uniquely Alters Kv4.3 Channel Gating

**DOI:** 10.1371/journal.pone.0003773

**Published:** 2008-11-20

**Authors:** Matthew R. Skerritt, Donald L. Campbell

**Affiliations:** Department of Physiology & Biophysics, School of Medicine and Biomedical Sciences, University at Buffalo, State University of New York, Buffalo, New York, United States of America; University of Cincinnati, United States of America

## Abstract

The S4 transmembrane domain in Shaker (Kv1) voltage-sensitive potassium channels has four basic residues (R1–R4) that are responsible for carrying the majority of gating charge. In Kv4 channels, however, *R1* is replaced by a neutral valine at position 287. Among other differences, Kv4 channels display prominent closed state inactivation, a mechanism which is minimal in Shaker. To determine if the absence of *R1* is responsible for important variation in gating characteristics between the two channel types, we introduced the V287R mutant into Kv4.3 and analyzed its effects on several voltage sensitive gating transitions. We found that the mutant increased the voltage sensitivity of steady-state activation and altered the kinetics of activation and deactivation processes. Although the kinetics of macroscopic inactivation were minimally affected, the characteristics of closed-state inactivation and recovery from open and closed inactivated states were significantly altered. The absence of *R1* can only partially account for differences in the effective voltage sensitivity of gating between Shaker and Kv4.3. These results suggest that the S4 domain serves an important functional role in Kv4 channel activation and deactivation processes, and also those of closed-state inactivation and recovery.

## Introduction

The S4 transmembrane domain has been shown to play an important role in the voltage sensitivity of Kv1 (Shaker-like) potassium channels [1]–[3]. The first four arginine residues (R1–R4) in S4 bestow voltage sensitivity and are responsible for carrying the majority of gating charge [1]–[5]. This mechanism has been assumed to underlie voltage-sensitive gating in Kv4 (Shal-type) channels as well, which generate rapidly activating and inactivating K^+^ current phenotypes designated “I_A_” in neurons and “I_to,fast_” in cardiac myocytes [6]–[8]. However, only recently has experimental evidence been obtained on the roles of positively-charged residues in S4 with respect to regulating Kv4 channel gating transitions [9], [10].

These previous studies, which eliminated individual S4 positive charges in Kv4.3 by mutation to uncharged alanine (R→A, [9]) or glutamine (R→Q, [10]), found that activation and deactivation characteristics were altered in a manner consistent with S4 serving a primary functional role of the voltage sensor domain, VSD. However, these mutants (which perturbed both electrostatic and structural properties of the native VSD) significantly altered closed state inactivation (CSI) and recovery from both open and closed inactivated states, effects that cannot be accounted for by the conventional Shaker model [11]–[13]. Specifically, Kv1 channels lack significant CSI [13], [14; however], [see 15], while the process is prominent in Kv4 channels [6]–[10], [16].

Although the mechanistic details of Kv4 channel inactivation gating are poorly understood, it is accepted that conventional Shaker N- and P/C-type mechanisms are not operative [6], [8], [16]. Also, with regard to activation, it has been noted that the steepness of the steady-state activation curve (“a^4^”) is ∼2–3 times less than that of Kv1 channels [8]–[10], [17]. While several factors likely contribute to these unique voltage sensitivities [1]–[5], [12], [18]–[20], an immediately obvious difference between the two channel types exists in the number of putative gating charges in S4: Kv1.4 has four (R1–R4), while Kv4.3 has three (R2–R4 using the previous nomenclature), with *R1* replaced by neutral and hydrophobic valine at position 287.
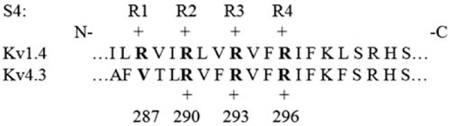



With prior studies providing evidence that S4 arginine residues at positions 290, 293, and 296 confer voltage sensitivity to multiple gating transitions in Kv4.3 [9], [10], we hypothesized that the absence of *R1* may account, to a degree, for noted differences in gating and regulatory characteristics between Shaker and Kv4 channels [6]–[8], [16]. To test this, we mutated the native residue at position 287 to arginine (V287R), a perturbation that introduced *R1*-like positive charge as well as expanded associated side chain volume by roughly 36 cm^3^/mole and increased local hydrophilic character [21], [22].

Here we demonstrate that V287R increased the steepness of the steady state activation curve and slowed activation while accelerating deactivation kinetics. The mutant also significantly altered the characteristics of CSI and recovery from inactivation. Our results suggest that the absence of *R1* only partially accounts for noted differences in voltage dependent gating characteristics between Shaker and Kv4.3; additional structural differences between the wild type and V287R mutant channels are likely involved. We also show that the mutant significantly accelerates recovery kinetics from both open-inactivated and closed-inactivated states, findings that further suggest that recovery is coupled to deactivation [8]–[10], [16], [23]. Non-inactivated closed states are stabilized in the mutant channel, consistent with S4 importantly regulating not only activation and deactivation processes, but also those of CSI and recovery. Our results support the proposal that CSI possesses inherent voltage dependence or is coupled to activation in a manner significantly different from that existing in Kv1 channels.

## Methods

### Mutagenesis

Kv4.3 was cloned from ferret heart (long form, GenBank AF454388) as described previously [10] and maintained in the pBluescript KS(+) vector. Site directed mutagenesis was performed using the Quick Change II Site-Directed Mutagenesis Kit (Strategene, La Jolla, CA, USA) and primers designed to valine 287 (Invitrogen, Carlsbad, CA, USA) in the fourth transmembrane segment. Specificity of mutations was confirmed by sequencing.

### In vitro Transcription and Oocyte Preparation

Kv4.3 wild type and mutant clone plasmids were linearized with the restriction endonuclease XhoI (New England BioLabs, Ipswich, MA, USA). cRNA was synthesized by the mMessage mMachine T7 Ultra Kit (Ambion, Austin, TX, USA). cRNA quantity and quality was evaluated by spectroscopy and agarose gel electrophoresis.

All animal protocols were conducted according to the NIH-approved guidelines of the Institutional Animal Care and Use Committee, University at Buffalo, SUNY. Oocytes were obtained from mature female *Xenopus laevis* euthanized by soaking in 6.0 g L^−1^ ethyl-3-aminobenzoate methanesulfonate salt and defolliculated as previously described [10]. Twelve to 24 hours after isolation, oocytes were injected with 4–9 ng cRNA (Nanoject II; Drummond Scientific, Broomall, PA, USA). Injected oocytes were incubated for 2–4 days at 18°C.

### Electrophysiology

Two-microelectrode voltage clamp (TEVC) recordings (GeneClamp 500B, Axon Instruments, Union City, CA, USA) were performed on injected oocytes as described previously [10]. Previous WT Kv4.3 data [9], [10], [16], [25] used for comparison were acquired in each study from a minimum of three different batches of oocytes. Recordings (22±2°C) were conducted in ND96 solution (in mM: 96 NaCl, 2 KCl, 1 MgCl_2_, 1.8 CaCl_2_, 5 HEPES, pH = 7.40). All voltage clamp recordings were conducted at the maximal gain of the amplifier (10,000×) and clamp rise time stability settings of 60–120 µs. Currents were acquired (filtered at 1 kHz, digitized at 5 kHz) with a Digidata 1320A 16-bit acquisition system under pCLAMP 9 software control (Axon Instruments).

### Protocols

**Figure 1:**
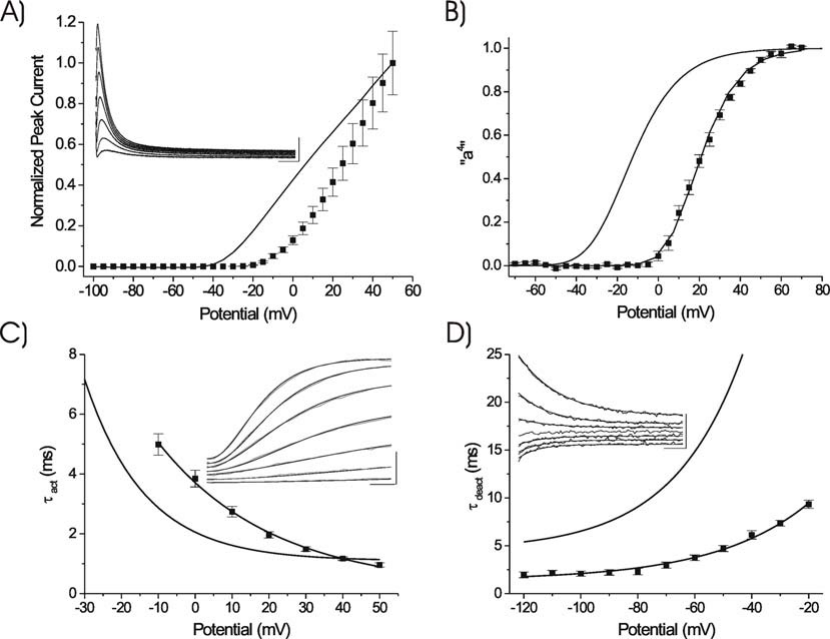
Activation and Deactivation Characteristics. A) *Main Panel*: Comparison of normalized (peak values at +50 mV) mean peak transient I–V relationships for V287R (n = 8) and WT Kv4.3, from [16]. HP = −100 mV, currents elicited by one second depolarizing pulses, and mean peak transient current defined as peak current minus residual current at the end of the depolarizing pulse. V287R mean peak current amplitude at +50 mV = 903±141 nA. *Inset*: Representative current waveforms for V287R elicited in response to depolarizing voltage clamp pulses applied from −10 mV to +50 mV, 10 mV increments. Calibration bar: 200 nA, 50 ms. B) Mean steady-state activation curve “a^4^” fit as a fourth order Boltzmann relationship (V_1/2_ = 1.40 mV, k = 11.62 mV, n = 11). Mean WT “a^4^” relationship: V_1/2_ = −36 mV, k = 14.50 mV, from [9]. C) *Main Panel*: V287R activation kinetics (n = 11). Curve fits (single exponential functions) to mean data points: τ_act_ = 4.84e^10−mV/30.9^+0.20 ms. *Inset*: Representative fits of V287R “a^4^” activation kinetics elicited during depolarizing pulses from −10 to +50 mV, 10 mV increments, HP = −100 mV. τ_act_ = 1.1, 1.4, 1.9, 2.5, 4.2, 5.2, 7.7 ms, −10 to +50 mV, respectively. Calibration bar: 200 nA, 1.0 ms. D) *Main Panel*: V287R deactivation kinetics (n = 12). Curve fits (single exponential functions) to mean data points: τ_deact_ = 0.18e^mV/32.7^+1.40 ms. *Inset*: Representative single exponential fits of V287R deactivation kinetics from −40 to −100 mV, 10 mV increments, HP = −100 mV. τ_deact_ = 4.9, 3.9, 3.6, 2.7, 2.8, 2.2 ms, −40 to −100 mV, respectively, −70 mV not fit. Calibration bar: 50 nA, 3.0 ms. WT data in panels C) and D) from [9].

Analysis of activation kinetics was conducted using the 90% voltage clamp rise time criteria employed in previous studies [9], [10]. A mean voltage clamp 90% rise time of τ_90%_ = 1.58±0.05 ms was obtained (n = 11). Fits to activation kinetics were only attempted after this time, so very rapid gating transitions that may have occurred during the initial voltage clamp rising phase were not resolvable. No quantitative claims regarding activation or deactivation kinetics prior to τ_90%_ are made. Estimates of minimal effective gating charges and voltage independent free energy changes were made using a simplified two state gating model [10], [20]. Briefly, at 22°C the movement of one elementary charge (e_0_) across the entire membrane potential field (δ_i_ = 1.0) would correspond to a slope factor (for either steady-state activation “a^4^” or inactivation “i”) of k = 25.43 mV. From fits to the experimentally measured “a^4^” and “i” curves (Figures 1.B, 2.A) the following estimates were made: the minimal effective gating charge q = RT/k, and the voltage independent change in free energy 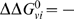, where V_1/2_ is the potential (mV) of half maximal activation or inactivation, k is the associated slope factor (mV), R is the gas constant, and T is temperature (° K). Kinetic estimates of q were obtained from exponential fits to the voltage dependence of the associated time constant curves. All data points in figures are mean±SEM values. Statistical significance (p≤0.01) was determined by ANOVA (Origin).

**Figure 2:**
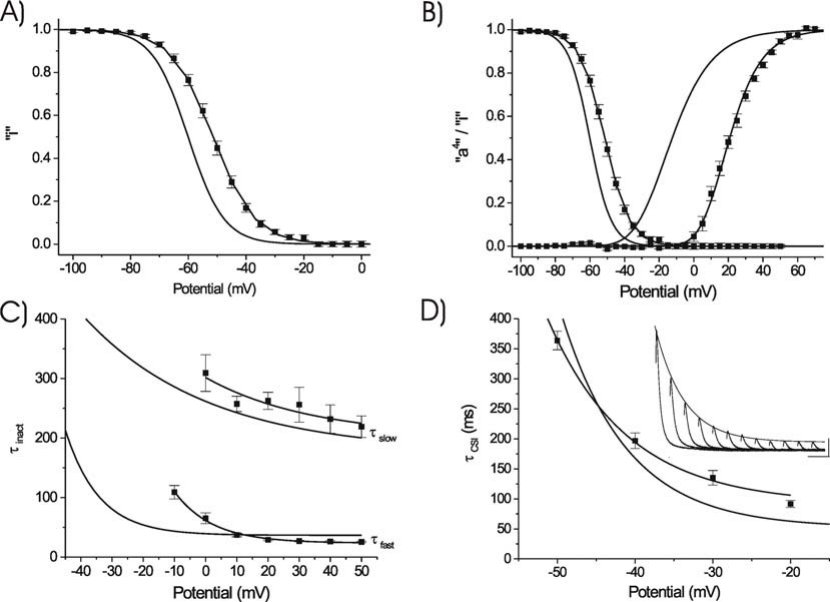
Inactivation Characteristics. A) Isochronal one second inactivation relationship “i”. Mean data points (n = 13) fit to a single Boltzmann relationship (V_1/2_ = −51.50 mV, k = 7.26 mV). WT data (V_1/2_ = −60.10 mV, k = 6.20 mV) from [9]. B) Comparative overlays of “a^4^” and “i” relationships for WT and V287R channels. Both expression conditions displayed significant closed-state inactivation, with V287R stabilizing non-inactivated closed states. The mutant channel also produced a greater depolarizing shift in “a^4^” than in “i”. C) V287R mean τ_inact_−V_m_ curves (n = 12). Curve fits: τ_fast_ = 86.20e^10−mV/12.23^+23.50 ms; τ_slow_ = 99.70e^10−mV/33.97^+201.90 ms. WT data from [10]. D) *Main Panel*: V287R kinetics of closed-state inactivation. A fixed one second pulse to +50 mV (P2) was preceded by a pulse of progressively increasing duration (P1) at each of the potentials indicated. The decline of P2 current as a function of P1 duration was used to determine τ_csi_. V287R curve fit: τ_csi_ = 725.68e^−60+mV/10.23^+91.50 ms. WT data from [25]. *Inset*: Development of CSI at −40 mV for V287R, τ_csi_ = 202 ms. Calibration Bar: 100 nA, 150 ms.

### Potential Limitations

Addition of a single charged residue to S4 and associated increases in “a^4^” and “i” slope factors, while suggestive, do not prove that the residue contributes to voltage sensitivity. Such a conclusion requires verification by appropriate gating current measurements and demonstration of alterations in single subunit gating charge [4], [5], [18]. We acknowledge the limitations of the two-state model, as previously discussed in detail [10, and references cited therein]. Further, there are several differences in uncharged amino acid residues between the S4 segments of Shaker and Kv4.3 (see *Introduction*), any of which may potentially contribute to the unique voltage sensitivities between the two channel types [24].

## Results

For reference, all measurements associated with WT Kv4.3 were acquired previously [9], [10], [16], [25], unless otherwise indicated. In associated figures, fits to this previous data are illustrated as smooth curves lacking data points.

### Activation and Deactivation Characteristics

Mean peak transient I–V relationships for WT and V287R are illustrated in Figure 1.A. We have previously observed an apparent activation threshold of ∼−40 mV for WT channels. In contrast, V287R resulted in a depolarizing shift, with an apparent activation threshold of ∼−15 mV.

To quantify this effect, we employed a saturating tail current protocol to directly estimate the steady-state activation relationship “a^4^”, fit as a standard Boltzmann relationship (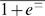) raised to the fourth power. Consistent with the depolarized I–V relationship, V287R produced a depolarizing shift in the mean half activation potential of ΔV_1/2_ = +37 mV (Figure 1.B). There was also an increase in the steepness of the “a^4^” curve (WT: k = 14.50 mV, V287R: k = 11.62 mV). These parameters gave the following estimates for a single α subunit: i) WT: q_act_ = 1.76 e_0_, V287R: q_act_ = 2.19 e_0_, an increase of 25%, and ii) a change in the voltage-independent free energy of activation of ΔΔG^0^_vi_ = 2.69 RT. V287R thus increased apparent effective q_act_ while stabilizing non-inactivated closed states.

Consistent with the depolarizing shift in “a^4^”, V287R slowed the kinetics of activation over the range of potentials hyperpolarized to 40 mV (Figure 1.C). Incorporation of *R1* also reduced the voltage-dependence of the τ_act_−V_m_ curve (as determined from an exponential fit to mean data points). A kinetic estimate of q_act_ = 0.82 e_0_ was obtained, a reduction of ∼50% from the WT value of 1.56 e_0_.

Deactivation kinetics (single exponential fits) were determined over a range of hyperpolarized potentials where mean activation curves indicated minimal measurable open state activity (WT: −40 to −120 mV, V287R: −20 to −120 mV). V287R significantly accelerated the kinetics of deactivation (Figure 1.D) while reducing the voltage-dependence of the τ_deact_−V_m_ curve. The latter effect resulted in a ∼25% reduction of effective q_deact_ (WT: q_deact_ = 1.04 e_0_, V287R: q_deact_ = 0.78 e_0_).

### Inactivation Characteristics: Kinetics of Development

The effects of V287R on the mean one second isochronal inactivation relationship “i” are illustrated in Figure 2.A (fit as a single Boltzmann relationship). In contrast to the effects on “a^4^”, V287R decreased the voltage sensitivity of “i” (WT: k = 6.20 mV, V287R: k = 7.26 mV), resulting in a 15% reduction in apparent effective q_csi_ (WT: q_csi_ = 4.10 e_0_, V287R: q_csi_ = 3.50 e_0_). Nonetheless, similar to WT, there was no significant overlap in the V287R “a^4^” and “i” relationships (Figure 2.B), indicating that a prominent closed state inactivation (CSI) mechanism was still present in the mutant channel.

The depolarizing shift in the half inactivation potential (ΔV_1/2_ = 8.60 mV) indicated that V287R stabilized non-inactivated closed states. Within our analytical framework (see *Methods*), this effect was attributed to perturbation of structural properties at the *R1* site, resulting in a change in the voltage-independent free energy of CSI of ΔΔG^0^_vi,csi_ = 2.60 RT. It was noted that the depolarizing shift produced by V287R as compared to WT was less for “i” than for “a^4^” (Figure 2.B).

Consistent with previous studies on WT channels [9], [10], [16], [25], the macroscopic inactivation kinetics of V287R (Figure 2.C) during a one second depolarizing pulse (applied from 0 to +50 mV) could be well described as a double exponential process (a single exponential time constant could only be obtained reliably hyperpolarized to −10 mV). The mean effects of V287R were subtle, with a slowing of τ_fast_ evident in the hyperpolarized range of potentials, an acceleration of τ_fast_ at more depolarized potentials, and a modest slowing of τ_slow_ across all potentials analyzed (Figure 2.C). The mean relative amplitude of the initial component of fast inactivation (A_fast_) was 0.85±0.01, a value similar to WT (A_fast_ = 0.80±0.02). Apparent voltage sensitivity of the τ_fast_−V_m_ curve was decreased in the mutant channel (WT: q_inact,fast_ = 2.36 e_0_, V287R: q_inact,fast_ = 2.08 e_0_), while for the τ_slow_−V_m_ curve it was increased (WT: q_inact,slow_ = 0.64 e_0_, V287R: q_inact,slow_ = 0.75 e_0_). Despite differences in these values, V287R produced no obvious alteration in macroscopic inactivation kinetics at potentials where the channel was nearly or fully activated.

In contrast to the minimal effects on the kinetics of macroscopic inactivation (Figure 2.C), it was predicted that V287R would alter the kinetics of development of CSI over a range of hyperpolarized potentials where the “i” curves of both WT and V287R were variable (Figure 2.A). To test this prediction, comparative τ_csi_−V_m_ relationships (−50 to −20 mV) were determined (Figure 2.D). The kinetics of the development of CSI displayed an exponential dependence upon potential. Depolarized to −40 mV, V287R slowed the development of CSI and reduced its apparent voltage dependence (WT: q_csi_ = 3.01 e_0_, V287R: q_csi_ = 2.49 e_0_).

### Inactivation Characteristics: Kinetics of Recovery

**Figure 3:**
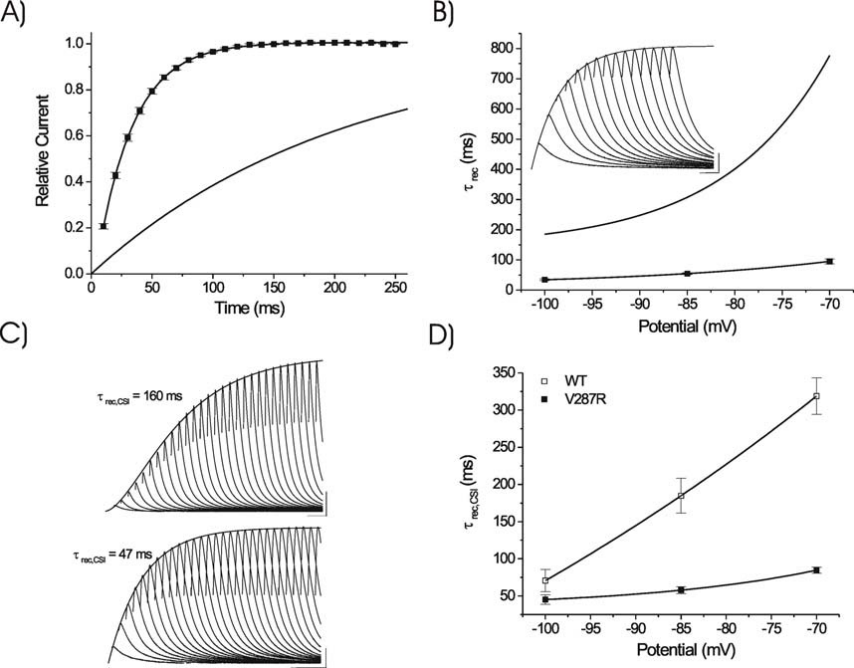
Recovery Characteristics. A) WT and V287R macroscopic recovery kinetics (HP = −100 mV) developed during a one second pulse to +50 mV. Mean mutant data points (n = 7) fit as a single exponential function with τ_rec_ = 30.10 ms. WT τ_rec_ = 206 ms, data from [9]. B) *Main Panel*: Comparison of the voltage dependence of mean τ_rec_ values of V287R and WT, from [9], [10], [16], [25]. At all HPs, macroscopic recovery kinetics were significantly faster for V287R than for WT. *Curve fits*: WT: τ_rec_ = 23.89e^mV/11.14^+142.10 ms, V287R: τ_rec_ = 9.42e^mV/21.77^+13.95 ms. *Inset*: Representative macroscopic recovery waveforms for V287R (P2 currents at +50 mV). Peak data points fit with a single exponential relationship with time constant = 32 ms. Calibration bar: 200 nA, 20 ms. C) Representative recordings of recovery (HP = −85 mV) from closed-state inactivation for WT (*upper panel*, fit as an “a^2^” formulation) and V287R (*lower panel*, exponential fit). Calibration bars: WT: 1 µA, 50 ms, V287R: 500 nA, 50 ms. D) Voltage dependence of mean τ_rec,csi_ values for WT (hollow squares, n = 5) and V287R (solid squares, n = 7). *Curve fits*: WT: τ_rec,csi_ = 248.50e^mV/93.1^−582.10 ms, V287R: τ_rec,csi_ = 5.80e^mV/19.77^+34.10 ms.

V287R significantly altered the kinetics of recovery from inactivation. Using a double pulse protocol (see [25]) mean recovery kinetics at HP = −100 mV (single exponential fits) were significantly faster for V287R than for WT (WT: τ_rec_ = 185 ms, V287R: τ_rec_ = 30.10 ms, Figure 3.A). This acceleration could not be attributed to a simple shift in the isochronal inactivation curve, as “i” = 1.0 at HP = −100 mV for both channel constructs.

At the holding potentials analyzed (−100, −85, −70 mV) the kinetics of recovery from macroscopic inactivation were always faster for V287R than for WT (Figure 3.B). The voltage dependence of the mean mutant macroscopic recovery time constant, τ_rec_, gave an estimated effective charge of q_rec_ = 1.16 e_0_, while previous results yielded a mean WT value of q_rec_ = 2.28 e_0_ [9], [10].

We have observed that recovery from closed-state inactivation (HP = −100 mV, developed during a two second P1 pulse to −50 mV) for the WT channel is a sigmoidal process that can be empirically fit as an “a^2^” exponential formulation [9]. To expand upon these observations, we applied the same protocol at HP = −85 and −70 mV to determine the voltage dependence of the time constants. Recovery from CSI for the WT channel was again sigmoidal at each HP and empirically fit as an “a^2^” formulation (Figure 3.C, *upper panel*). In contrast, recovery from CSI for V287R could be well fit as a conventional exponential process (Figure 3.C, *lower panel*). For both channels, as HP was depolarized, CSI recovery kinetics were slowed (Figure 3.D). Nonetheless, V287R displayed significantly faster kinetics than WT at each HP, and increased the apparent voltage dependence of the τ_rec,csi_ curve (WT: q_rec_ = 0.27 e_0_, V287R: q_rec_ = 1.29 e_0_).

## Discussion

In this study we found that incorporation of a single arginine residue at position 287 in Kv4.3 increased the steepness of the steady-state activation relationship and its associated effective q_act_ value. These findings complement previous work indicating that elimination of S4 native charge at positions 290 and 293 reduced the voltage sensitivity of “a^4^” [9], [10]. However, despite an increase in the steepness of the steady-state activation curve as compared to WT, the mean slope factor for V287R was still greater (less voltage sensitive) than that reported for Kv1 channels. Therefore, the absence of *R1* charge alone can only partially account for noted differences in activation characteristics between Kv1 and Kv4.3 channels. Effects resulting from perturbation of structural characteristics must also be considered. For example, in addition to conferring supplementary positive charge to S4, the V→R mutation expanded associated side chain volume by ∼36 cm^3^/mole and introduced local hydrophilic character. Overall effects on activation characteristics were thus likely due to alteration of both electrostatic and structural properties [10], [20], a finding in agreement with prior studies on Kv1 channels [26].

**Figure 4:**
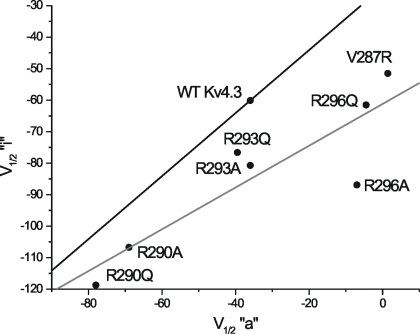
Summary of all Kv4.3 S4 mutant data collected to date: “i” V_1/2_ values as a function of “a” V_1/2_ values. The solid black line (centered on WT) is a linear relationship with slope = 1.0, as predicted from previous work on Shaker channels [26]. The solid gray line is a best fit to all mutant data points (WT excluded, slope = 0.65, mean R→A/Q data points from [9], [10]). All ΔV_1/2_ shifts were less than those predicted from Shaker.

The effects of V287R on closed state inactivation (CSI) characteristics were not predictable from the Shaker model, as minimal CSI is displayed by Kv1 channels [13], [14], and apparent voltage dependence of inactivation arises from coupling to activation [19]. While depolarizing shifts in both “a^4^” and “i” produced by V287R are consistent with partial coupling of inactivation to activation, we have demonstrated previously that charge neutralization of specific arginine residues in S4 can produce non-parallel, and even opposite, shifts in “a^4^” and “i” [9], [10]. This suggests that CSI can be uncoupled from activation. In Shaker channels, Papazian et al. [26] originally demonstrated that the S4 mutants that altered the voltage-dependence of activation also altered inactivation to similar extents, and the relationship between the V_1/2_ values of activation and inactivation was linear with a slope close to 1.0. For comparison, a plot of all presently available Kv4.3 S4 mutant data is illustrated in Figure 4. A linear relationship centered on WT and with a slope of 1.0 could not adequately describe our results, with all V_1/2_ shifts less than those predicted by Shaker. In addition, V287R increased the voltage sensitivity of “a^4^” while reducing it in “i”.

Previously, we have analyzed the effects of S4 R→A mutant channels and observed that R290A, R293A, and R296A produced variable and non-parallel effects on “a^4^” and “i”, and each significantly slowed macroscopic recovery and deactivation processes [9]. In comparison, we report here that V287R depolarized “a^4^” and “i” (again in a non-parallel fashion), and significantly accelerated macroscopic recovery and deactivation kinetics. Taken together, these studies suggest that S4 positively charged residues are importantly involved in regulating several unique gating transitions in Kv4.3, in particular CSI and recovery.

R302A (corresponding to Shaker R5 and localized to the intracellular half of the S4 domain) was also analyzed in that study, and found to produce effects similar to V287R. Specifically, both mutants depolarized the steady-state activation relationship. Although R302A “a^4^” could not be measured directly (explanation provided in [9]), its mean peak I–V relationship was similar to V287R, with an activation threshold near −15 mV. The kinetics of deactivation and macroscopic recovery were also accelerated in both mutants. However, although V287R and R302A both depolarized the steady state inactivation relationship and reduced voltage sensitivity of inactivation (V287R: k = 7.26 mV, q_csi_ = 3.50 e_0_; R302A: k = 7.50 mV, q_csi_ = 3.40 e_0_), the depolarizing shift in “i” was much greater for R302A (21.4 mV) than for V287R (8.6 mV). This suggests that addition of a putative gating charge at position 287 and its elimination at position 302, while yielding superficially similar effects, do so by distinctly different mechanisms. Nonetheless, it is interesting to note that opposing mutations at opposite ends of the S4 domain can result in a similar gating phenotype.

In contrast to Kv1 N-type inactivation [13], [14], [19], we propose that Kv4.3 CSI possesses inherent voltage dependence. Partial N-terminal deletion does not alter CSI characteristics in Kv4.2 (Δ2-40, [27]) or Kv4.3 (Δ2-39, unpublished observations). Therefore, if apparent voltage sensitivity of Kv4.3 CSI does arise from partial activation of non-conducting closed states (early gating transitions that precede the final closed-to-open state), a Kv1-like N-terminal inactivation domain cannot be a primary inactivation mechanism.

The closed state structure of any voltage-sensitive potassium channel has yet to be solved. As a result, all existing closed state models are speculative and based on Kv channels that display minimal CSI [28]–[30]. This is important to note considering that Kv1 channel gating current measurements propose that the register of S4 may be significantly different between open and open inactivated states [31]. Our data indicate that a similar scenario likely exists in Kv4.3 non-inactivated closed versus inactivated closed states. Campos et al. [28] have proposed that in the closed state, Shaker R1 is positioned in the outer half of the membrane, oriented toward S1–S3 and in close proximity to I241 in S1 and I287 in S2. These residues, which in Kv4.3 correspond to I198 in S1 and I236 in S2, may form a hydrophobic septum separating the extracellular and intracellular crevices of the gating pore [1], [32], [33]. Alternatively, in a study of chimaeric Kv1.2–Kv2.1 channels, Long et al. [29] have proposed that phenylalanine 233 in S2, positioned three residues “down” from corresponding Shaker I287, forms the septum. Kv4.3 has a comparable phenylalanine residue at position 237 in S2.

During voltage dependent gating transitions, the hydrophobic septum is believed to focus the transmembrane electric field to a narrow region of S4 [34]–[36]. In the closed-state of Kv1, the field is believed to reside across R1 [3], [5]. Applying the model of Campos et al. [28] to Kv4.3 suggests that in the non-inactivated closed state the electric field would be focused over a region of S4 lacking positive charge. Our results indicate that insertion of non-native *R1* increases the voltage sensitivity of steady-state activation. We therefore propose that the outer crevice in WT Kv4.3 channels extends further into the transmembrane domain than it does in Shaker [10], thus allowing the field to be focused across R290 (R2 in Kv1) in the closed state. This proposal is consistent with the model of Long et al. [29]. Alternatively, the hydrophobic septum may be thicker in Kv4.3 than in Kv1. A thicker septum would unfocus the transmembrane field while still allowing it to influence R290, resulting in reduced voltage sensitivity. In both models [28], [29], insertion of *R1* may alter the field sensed by other positively-charged residues in S4. Gating current studies in Kv1 channels have demonstrated that specific S4 mutants can produce non-additive effects, demonstrating that such mutants can alter the voltage field sensed by other gating charges [37], [38].

The majority of the Shaker S4 mutants analyzed by Papazian et al. [26] failed to alter the kinetics of recovery. In contrast, all Kv4.3 R→A/Q mutants significantly altered recovery kinetics. Those that were found to stabilize closed inactivated states slowed the process, while those that stabilized non-inactivated closed states accelerated it [9], [10]. By accelerating recovery (by nearly an order of magnitude), we propose that V287R stabilizes non-inactivated closed states. These findings are comparable to those resulting from coexpression of Kv4.3 with KChIP2 isoforms [16], [25], [39]. Although V287R and KChIPs likely do not accelerate the process by the same mechanism, they do share a common element in that both also accelerate the kinetics of deactivation. These parallel effects further support the proposal that recovery and deactivation processes are coupled [8]–[10], [16], [25]. Although there is present controversy with respect to Kv4 channel gating models [7], [8], [17], [23], [40], Wang et al. [23] have suggested that the failure of all such models to predict the voltage dependence of recovery arises from their inability to account for the energetic coupling between deactivation and recovery. Our results support this proposal.

In conclusion, the results presented here indicate that the difference in the voltage dependence of activation between Kv1 and Kv4 channels cannot be fully accounted for by the absence of *R1* in Kv4.3. Likely, additional structural characteristics unique to the S4 transmembrane domain of each channel are involved. Additionally, demonstration that V287R significantly alters both macroscopic recovery kinetics and closed-state inactivation characteristics provides further evidence that the S4 domain not only mediates voltage sensitivity of Kv4.3 activation and deactivation processes, but also those of closed state inactivation and recovery.
